# Ecological, morpho-agronomical, and nutritional characteristics of *Sulla flexuosa* (L.) Medik. ecotypes

**DOI:** 10.1038/s41598-023-40148-y

**Published:** 2023-08-16

**Authors:** S. Boukrouh, A. Noutfia, N. Moula, C. Avril, J. Louvieaux, J. L. Hornick, M. Chentouf, J. F. Cabaraux

**Affiliations:** 1 Department of Veterinary Management of Animal Resources, Faculty of Veterinary Medicine, FARAH Center, University of Liège, 4000 Liège, Belgium; 2 Regional Center of Agricultural Research of Tangier, National Institute of Agricultural Research, 10090 Rabat, Morocco; 3 Haute École Provinciale de Hainaut Condorcet, Agronomy Category, 7800 Ath, Belgium

**Keywords:** Plant breeding, Plant domestication, Plant ecology

## Abstract

The present work was part of assessing wild genetic plant resources of forage interest in Northern Morocco and aimed to study the agro-morphology and nutritional value of *Sulla flexuosa* (L.) Medik. (*Hedysarum flexuosum* L.) ecotypes. The seeds of twenty-one wild *S. flexuosa* (L.) Medik. ecotypes were collected from 21 sites. The edaphic and climatic characteristics of the collection sites were studied and testified to the remarkable adaptability of *S. flexuosa* (L.) Medik. These 21 ecotypes were cultivated in three complete randomized blocks design for two consecutive years. Statistical analysis showed substantial variability between the collected ecotypes. Principal component analysis and heatmap analysis allowed to distinguish four groups of ecotypes mainly based on nutritional parameters (fiber content and digestibility), forage production (dry matter yield, number of leaves per plant, and total number of branches), and reproduction (number of inflorescences per plant and, weight of thousand seeds and seeds per plant). Furthermore, the present study pointed out the value of ecotype 1, which was dual purpose with its high productivity, nutritional value, and reproductive parameters. Ecotype 4 was also highlighted as having late flowering but intermediate productivity, which can be used mainly for haymaking as the drying period could coincide with the last rainfall in the region.

## Introduction

In the southern Mediterranean area, such as Northern Morocco, goat farming significantly contributes to rural household income. These farms are often familial, extensive, driven by traditional knowledge and know-how, and based on the exploitation of free natural resources^1^. The feeding of this extensive or semi-extensive livestock is characterized by a strong pressure on the silvopastoral rangelands and an irregular forage supply that does not meet animal requirements throughout the year^2^. To sustain forests and improve animal productivity, it is therefore necessary to study new fodder resources to secure forage systems^1,2.^

Endemic fodder legumes of natural pastures are essential elements in the diet of ruminants, among others, because of their high protein content.

Among the wild legumes found in the Mediterranean region, annual and perennial species of the genus *Hedysarum spp*. grow on a remarkable range of bioclimatic and soil conditions^3^. In addition, due to their moderate to high concentration of condensed tannins, species of the genus *Hedysarum spp*. exhibit antiparasitic effects in the digestive tract, a decrease in microbial degradation of proteins in the rumen with improved intestinal absorption, and positive environmental impacts by reducing methane production in ruminants^4^. The interest in the genus *Hedysarum spp*. also comes from the fact that some species have good agronomic characteristics and, in particular, excellent adaptability to marginal and dry environments^5^, low input requirement, and contribution to soil nitrogen enrichment through their ability to fix atmospheric nitrogen by symbiotic association with rhizobia^6^. They are nutritious and palatable legumes primarily used as a green forage for grazing and hay production, and in some regions of the Mediterranean area, they were also exploited as silage. Sulla is also a cover crop that improves soil fertility and reduces erosion^7^. In addition, its importance also lies in its versatility for its agricultural and non-agricultural uses. Sulla was used for honey production and landscape architecture^8^.

Among the species of the genus *Hedysarum spp*., *Hedysarum coronarium* L., also called *Sulla coronaria* (L.) Medik., Italian or Spanish sainfoin, is commonly cultivated in the Mediterranean basin^5,9^ and has been widely studied for its nutritional value^10^ and phenolic compounds^11^ at different morphological stages and through different conservation methods^12^. In Italy, some varieties of *S. coronaria* (L.) Medik (Grimaldi, Sparacia, Bellante, and S. Homer) were selected and are already cultivated and listed in the Italian national seed register.

In Morocco and Algeria, it is *H. flexuosum* L., also known as *S. flexuosa* (L.) Medik.^13^, which is encountered. This legume is a wild and neglected plant found in the wild natural grasslands in the form of small and isolated populations.

It is reported on marly and marl-limestone substrates in regions with average rainfall above 550 mm^3^. However, because of its spontaneous and non-cultivated character, *S. flexuosa* (L.) Medik. is classified on the IUCN’s red list of species at high risk of extinction^14^. Given the disappearance of several ecotypes in areas where they were once observed some years ago, this rarefaction could be accentuated by the extension of cultivation, soil degradation, overgrazing, or climate change. However, the conservation and use of this local plant resource could increase fodder supply and thus be introduced into the ruminant diet as an alternative to other legumes^15^. In Algeria, morphological and nutritional values of fresh and sun-dried *S. flexuosa* (L.) Medik. were assessed for rabbit fattening^16,17^. In Morocco, Errassi et al.^18,19^ reported significant variations in digestibility and phenolic compounds between *S. flexuosa* (L.) Medik. ecotypes; this first study was realized only for some parameters on a few ecotypes.

The present work aimed to deeply evaluate the ecological, agro-morphological, and nutritional characteristics of twenty-one *S. flexuosa* (L.) Medik. ecotypes in Northern Morocco. The aim was to create a seed bank for conservation and choose the more productive and interesting ecotypes for cultivation and animal nutrition.

## Material and methods

### Plant material, study site, and experimental set-up

#### Collection of ecotypes

**Figure 1:**
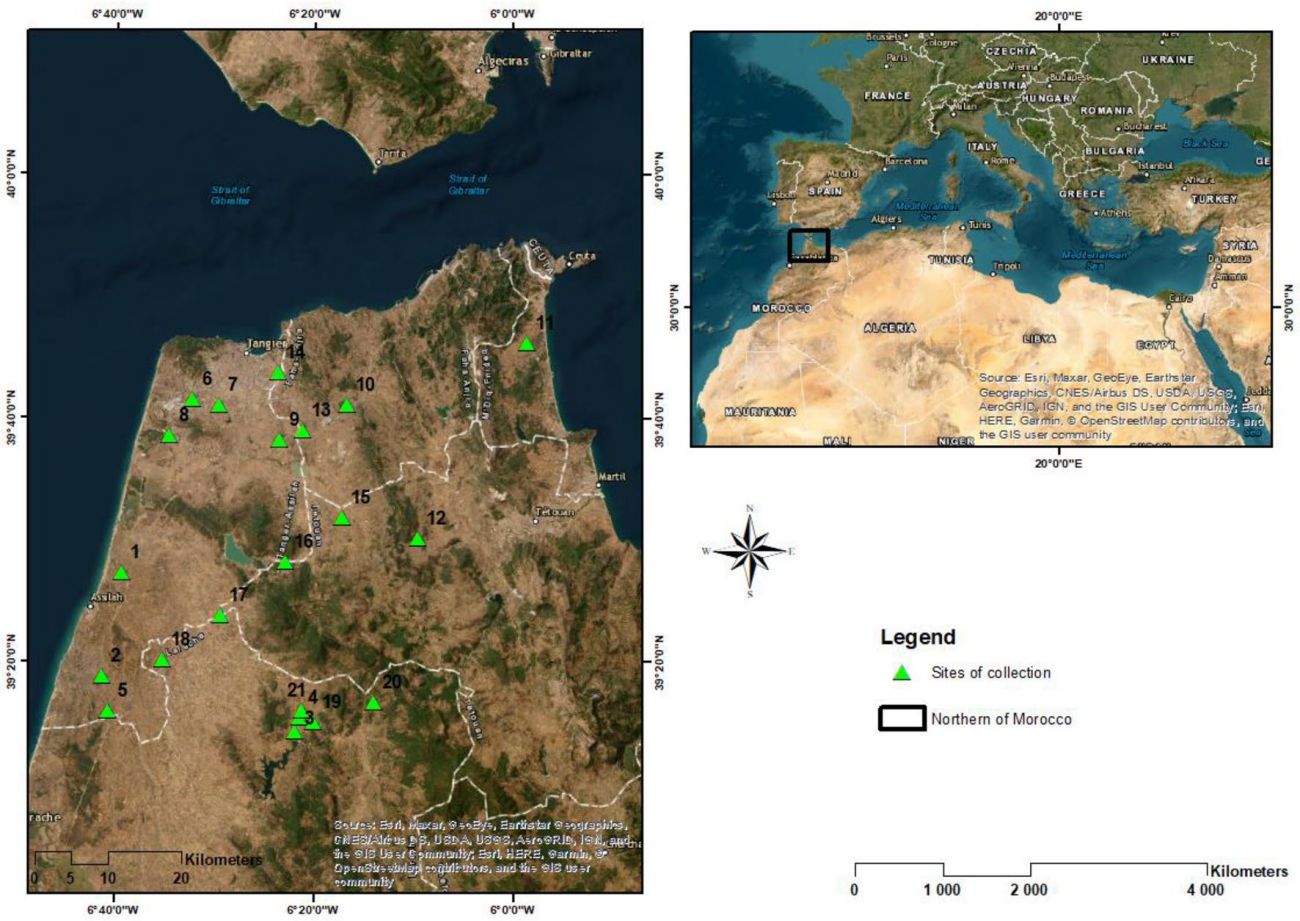
Collection sites of the 21 *S. flexuosa* (L.) Medik. ecotypes (This map was generated using ArcGIS Desktop version 10.4.1—Esri, Redlands, CA, USA, https://en.freedownloadmanager.org/Windows-PC/Portal-for-ArcGIS.html ).

The plant material used consisted of twenty-one *S. flexuosa* (L.) Medik. ecotypes collected on twenty-one sites in Northern Morocco in June 2018 (Fig. 1). They were collected using FAO protocole^20^ and according to the relevant institutional, national, and international legislation. Indeed, as the *S. flexuosa* (L.) Medik. is present on the IUCN red list, the Regional Direction of Agriculture of Tangier–Tétouan–Al Hoceima (Morocco) has authorized the National Institute of Agricultural Research (INRA) to collect seeds of local germplasms for a scientific objective of characterization and preservation. At each site, over 50–100 m^2^, mature pods were collected from a minimum of 30 random plants, collecting the maximum number of mature pods per plant. The seeds were kept at room temperature before sowing and then sent to INRA genebank for long-term storage. The seeds are available to seed exchange in research networks at a request addressed to the director.

#### Ecological characterization

Nineteen bioclimatic data (BIO 1–19) from the period 1950–2000 were estimated for each collection site from major climate databases (www.worldclim.org). The data were extracted from satellite images via ArcGIS Desktop version 9.3 (Esri, Redlands, CA, USA) and were interpolated with a resolution of approximately 1 km^21^. Climatic data (minimum, maximum, mean temperatures (°C), rainfall of each month, and total rainfall (mm) of the 2019 and 2020 agronomical years) of the experimental site were collected from a climatic station 10 km nearer. The bioclimatic variables of the two years were calculated based on these climatic data. At the collection sites, five samples of soil from the 0–20 cm and five from the 20–40 cm horizons were collected for edaphic analysis. Soil samples were oven dried at 60 °C until constant weight and humidity was calculated as the difference between wet and dry samples. They were ground and passed through a 2 mm sieve to remove larger particles. The pH (water and KCl) was read with a standard calibrated pH meter in 2:1 distilled water and dry soil ratio^22^. Electrical conductivity was measured in the soil extract collected from the saturated soil paste by conductivity meter^23^. Nitrogen was determined by mineralization and distillation using the Kjeldahl method^24^. Exchangeable potassium (K) was analyzed in 1 N ammonium acetate extract using a flame photometer^25^. Total limestone (CaCO_3_) was measured by treating the sample with HCl^26^. Carbon was measured through dichromate oxidation and converted to organic matter by multiplying by a factor of 1.72^27^. Available phosphorus (P) was obtained by the colorimetric method^28^. Soil texture was determined by using standard Pipette method and wet sieving^29^.

#### Experimental design

The study was set up during both the agronomic years 2018/2019 (2019) and 2019/2020 (2020) in El Menzla, a related field of Regional Agronomic Research Institute of Tangier (Morocco) (35° 31′ 53′′ N; 5° 42′ 36′′ W; 128.5 m AMSL). From the collected ecotypes, in greenhouse trays, 3 to 5 seeds were sowed per alveolus on November 1, 2018, and October 29, 2019. Four weeks after sowing, in the field, 60 seedlings were transplanted into a plot in a randomized complete block design with three replications (i.e., 180 seedlings per ecotype a year). In one plot, seedlings were transplanted in six 2-m rows with 30 cm between rows and 22 cm between plants. The distance between plots was 50 cm, and between blocks was 1 m. *Sulla flexuosa* (L.) Medik. seeds were not inoculated with rhizobium before sowing because prolific nodulation occurs naturally on this site. The trial was conducted under rainfed conditions on fallow plots during 2019 and 2020, and NPK (10-30-10) fertilizer was applied at the rate of 100 kg/ha on the day of transplantation of plants into the plots. Irrigation was applied one time on the same day to avoid nitrogen volatilization. Natural weed was removed manually during the growing season.

### Agro-morphological characterization

#### Phenological assessment

The number of days between sowing and emergence (NDE), appearance of the first flower bud (FBA), start of flowering (SF), full flowering (FF), and end of flowering (EF) were recorded during regular visits (every three days) to the experimental site in the first year. The flowering duration (FDUR) was determined by the difference between the start and the end of flowering. For the second year (2020), the confinement due to COVID-19 did not allow the phenological characterization. The different phenological stages were expressed in growing degree days (GDD) by considering the base temperature of 10 °C^30^. They were determined by the following formula “Eq. (1)”:

1 $${\text{Accumulated GDD}} \,({^\circ{\text{C}}}) = \Sigma \left[ {\left( {{\text{T}}_{{{\text{max}}}} + {\text{T}}_{{{\text{min}}}} } \right)/{2}} \right] - {\text{T}}_{{\text{b}}}$$

Where T_max_ is the daily maximum temperature (°C), T_min_ is the daily minimum temperature (°C), and T_b_ is the base temperature (10 °C).

#### Morphological assessment

At the start of flowering and the full flowering during the two years, five plants were selected for morphological characterization (number of plagiotropic branches (NPB), plant height (PH), length of the orthotropic axis (LOA), length of the longest plagiotropic branch (LLPB), number of leaves per plant (NLP), number of inflorescences per plant (NIP), number of total branches (NTB), stem diameter (SD).

#### Agronomical assessment

The fresh and dry matter yields (FMY and DMY) were determined at the budding, start of flowering, and full flowering stages. At each stage, 0.75 m^2^ per plot was harvested and weighed. The plants were cut at the height of 5 cm above the soil to avoid soil contamination. Then 20 g of fresh matter were dried at 102 ± 1.0 °C with forced ventilation until constant weight to estimate the DM content of the samples and thus the DMY. In addition, a second sample of 1 kg of fresh matter was used to separate the different constituents of the cut plants: leaves, stems, and flowers. The leaves and stems were then dried at 102 ± 1.0 °C with forced ventilation to a constant weight to calculate the leaf-to-stem ratio (LSR) on a DM basis. At the physiological maturity stage, five plants were harvested and threshed to determine seed weight per plant (SWP); one thousand seeds were weighed thrice to determine thousand seed weight (TSW).

### Nutritional characterization

#### Chemical composition

Whole plants of *S. flexuosa* (L.) Medik. harvested at budding, early flowering, and full flowering stages were used to determine FMY, DMY, and the nutritional value of the forage. Samples were oven-dried at 60 °C for 48h, then ground and sieved through a 1 mm sieve, and stored in a desiccator.

The AOAC^31^ methods were used for analyses. Ash content was determined after incinerating 2 g of dried samples in a muffle furnace at 550 °C for 12 h (No. 942.05). Fat content (FC) was obtained by the Soxhlet method using diethyl ether as a solvent (No. 963.15). Crude protein (CP) content was determined by multiplying nitrogen content by 6.25, obtained using the Kjeldahl method (No. 977.02). Fiber content [crude fiber (CF) and fibers NDF (Neutral Detergent Fiber), ADF (Acid Detergent Fiber) and ADL (Acid Detergent Lignin)] was analyzed using an ANKOM® 200 Fiber Analyser (ANKOM Technology, Macedon, NY, USA) (No. 962.09) for CF and the method of Van Soest et al.^32^ for NDF, ADF, and ADL. The nitrogen-free extract (NFE) was estimated using the following formula “Eq. (2)”:

2 $${\text{NFE}}\left( {\% {\text{ DM}}} \right) = {1}00 - \left( {{\text{CP}} + {\text{Fat}} + {\text{CF}} + {\text{Ash}}} \right)$$

Quantification of total phenols (TP) and total tannins (TT) was performed according to the procedure described by Makkar et al*.*^33^ Briefly, the extraction was done by mixing 10 mL of 70% acetone with 200 mg of finely ground dry sample. The mixture was subjected to ultrasonic treatment for 20 min and centrifugation for 10 min at 3500 rpm at 4 °C. In a tube, 0.05 mL of the extract was mixed with 0.25 mL of Folin Ciocalteu 1N reagent, 0.5 mL of distilled water, and 1.25 mL of 20% sodium carbonate solution. The solution was then vortexed and left in the dark for 40 min; the absorbance was read at 725 nm to determine total phenols. In another centrifuge tube, 3 mL of the extract was mixed with 300 mg of polyvinylpolypyrrolidone and 3 mL of demineralized water. The tube was vortexed, kept at 4 °C for 15 min, centrifuged at 4 °C at 3500 rpm for 10 min, and then read at 725 nm to determine non-tannic phenols (NTP). Total tannins were calculated as the difference between non-tannic phenols and total phenols. Condensed tannins (CT) were analyzed by Porter et al.^34^ method. Briefly, in a glass tube, 3 mL of Butanol-HCl reagent and 0.1 mL of ferric reagent were mixed with 0.5 mL of the extract. The tubes were vortexed and put in a water bath at 97 °C for one hour. After cooling, the absorbance was read at 550 nm. The difference between TT and CT deduced hydrolyzable tannins (HT).

#### Digestibility

The In Vitro Enzymatic CP Degradability (IVECPD) was determined by the method of Aufrère and Cartailler^35^. Briefly, in a centrifuge tube, 25 mL of a protease solution was added to 0.5 g of the sample and was incubated at 40 °C for one hour. The sample was centrifuged for 5 min at 3500 rpm and then filtered. The liquid phase was mineralized for one hour using sulfuric acid at 120 °C and for 2 h at 350 °C. The tube containing the mineralized sample was connected to the distiller. Finally, the contents of the Erlenmeyer flask were titrated with 0.1 N HCL to determine the digested nitrogenous matter.

The In Vitro True Digestibility (IVTD) was determined by incubation of feed samples in filter bags in a Daisy II incubator® (ANKOM Technology, Fairport, NY, USA)^36^. The rumen liquor was obtained from goats at a communal slaughterhouse. Rumen fluid was collected into a pre-warmed thermos and transported to the laboratory, where rumen fluid was purged under CO_2_ for 30 s. About 500 mg of each feed was placed in ANKOM F57 filter bags with a pore size of 57 mm (ANKOM, Macedon, NY), which were heat-sealed, and subsequently put in jars (24 bags/jar). The rumen liquor was added to artificial saliva in a 1:5 ratio, and then the mixture was added in jars and incubated at 39.5 °C for 48 h. The In Vitro Digestibility was estimated by quantifying residual DM compared to incubated initial quantities. The enzymatic pepsin-cellulase method was used to determine the enzymatic dry matter digestibility (IVEDMD) and enzymatic organic matter digestibility (IVEOMD) of *S. flexuosa* (L.) Medik.^37^, according to a two-step method: 0.5 g of dried sample was incubated at 40 °C with 20 mL of a 2% pepsin solution diluted in 0.1 N hydrochloric acid and shaken constantly for 24 h; then the sample was solubilized in 50 mL of a buffer solution containing 1 g/L cellulase and, shaken and incubated at 40 °C for 24 h. After incubation, the sample was rinsed with hot distilled water and placed in an oven at 60 °C until constant weight. It was weighed to determine the IVEDMD. The sample was incinerated in the muffle furnace at 550 °C for 12 h to determine IVEOMD.

The metabolizable energy (ME; MJ/kg DM) was calculated using the “Eq. (3)” of AOAC^31^:

3 $${\text{ME (MJ/kg DM)}} = 0.{17} \times {\text{IVEDMD}}{-}{2}$$

where IVEDMD is the enzymatic dry matter digestibility in percentage.

### Data analysis

Analysis of variance was carried out to test the years, ecotypes, phenological stages, and their interactions. The variance components were estimated using a general linear model (GLM), using SAS 9.4 version (SAS Inst. Inc., Cary, NC, USA). The phenological stage was considered as a fixed factor in the model. To study the correlations between the different parameters, a correlation matrix was created based on Pearson’s correlation coefficients using the “corrplot” R software (Version 4.2.1). Principal component analysis (PCA) was performed for all parameters using the “Factominer and Factoextra” packages of R software. Only variables that were well described on the axes were retained. A heatmap summarising all the morphological, agronomical, and nutritional parameters contributing to variation between *S. flexuosa* (L.) Medik. ecotypes was created using the “Pheatmap” package of R software, with Euclidean distance as the similarity measure and hierarchical clustering with complete linkage.

## Results and discussion

### Ecological characterization

In Northern Morocco, the wild-collected *S. flexuosa* (L.) Medik. ecotypes were found on sites with an altitude not exceeding 358 m and minimum annual rainfall of 661 mm (Table 1). The physico-chemical characteristics of the soils were variable from one site to another. However, the results showed that these soils were rather dry (5% moisture), basic (pH = 8.4), and poor in organic matter (2%), exchangeable potassium (137 ppm), and available phosphorus (9.6 ppm). The average electrical conductivity of the soil (9.8 mS/m) was below 20 mS/m and corresponded to a non-saline soil, according to Boulding et al.^38^. The limestone content did not exceed 20%. These results are close to those reported by Abdelguerfi-Berrekia et al.^3^ and Zirmi-Zembri and Kadi^16^ with a minimum rainfall of 550 mm, a maximum altitude of 600 m, a pH ranging from 7.4 to 8.9, an organic matter (OM) content ranging from 0.21 to 2.54%, and a CaCO_3_ lower than 20%. However, these authors reported rather clayey or sandy soils on the collection sites of wild *S. flexuosa* (L.) Medik. In contrast, Yemlahi et al.^39^ found *S. flexuosa* (L.) Medik. in clay-loam soils. In the present study, the soil contained a minimum of 11% clay, while for sand and silt, the minimum percentages could reach 0%; the soil must apparently contain a minimum of clay for *S. flexuosa* (L.) Medik. growth. A high content of coarse silt (76%) was observed at one collection site. All these results show the high adaptability of *S. flexuosa* (L.) Medik. to grow on very different soils in textural composition.

**Table 1:** Climatic and physico-chemical characteristics of the soils of the 21 collection sites of wild *S. flexuosa* (L.) Medik. ecotypes and of the experimental site of *S. flexuosa* (L.) Medik. cultivation in Northern Morocco.

	Collection sites							Experimental site	
	Mean	Minimum	Maximum	Standard deviation	1st quartile	Median	3rd quartile	2019	2020
Altitude (m)	120.1	9.0	358.0	85.4	69.0	106.0	143.0	128.5	110.4
Edaphic parameters									
Humidity (%)	5.0	1.6	7.2	1.4	4.2	5.2	5.8	5.2	6.4
pH water	8.4	7.4	8.9	0.3	8.3	8.5	8.6	8.4	8.3
pH KCl	7.7	6.9	8.0	0.3	7.5	7.7	7.7	7.4	7.2
Electrical conductivity C (mS/m)	9.8	6.7	11.8	1.2	8.9	9.8	10.5	31.0	39.0
Organic matter (%)	1.9	0.6	3.7	0.9	1.3	2.0	2.5	2.2	2.6
Limestone (CaCO_3_, %)	5.1	0.5	20.3	5.5	2.0	3.2	5.3	7.4	3.9
Exchangeable potassium (K, ppm)	136.5	53.8	319.6	70.4	84.5	122.9	169.0	705.3	367.7
Available phosphorus (P_,_ ppm)	9.6	4.1	11.9	1.7	8.8	9.6	10.4	39.7	28.0
Carbon to nitrogen ratio (C/N)	11.7	2.6	24.5	5.8	6.6	9.2	15.4	6.4	8.0
Nitrogen (N, %)	0.1	0.1	0.2	0.1	0.1	0.1	0.2	0.2	0.2
Clay (%)	30.0	11.0	52.0	13.6	19.7	29.2	41.5	50.0	30.0
Coarse sand (%)	8.2	0.1	40.0	11.1	1.3	2.1	13.2	1.6	2.0
Fine sand (%)	4.4	0.2	13.7	4.4	1.5	2.5	5.6	1.1	0.9
Coarse silt (%)	38.6	0.0	75.6	22.5	18.1	45.2	46.2	35.0	34.0
Fine silt (%)	18.9	0.0	50.0	12.6	10.7	20.0	24.0	10.0	10.0
Bioclimatic parameters									
Average annual temperature (BIO1, °C)	18.1	17.3	18.4	0.3	18.1	18.2	18.2	18.1	18.5
Average diurnal variation (BIO2, °C)	8.8	6.0	10.0	1.1	8.2	9.1	9.7	22.9	20.4
Isothermality (BIO3 = BIO2/BIO7 × 100, °C)	39.1	33.1	41.1	2.1	38.5	39.8	40.6	57.3	51.1
Temperature seasonality (BIO4, %)	480.1	439.5	548.2	29.0	454.6	478.7	503.3	542.1	520.4
Maximum temperature of the warmest month (BIO5, °C)	29.0	27.9	29.9	0.7	28.4	29.1	29.7	40.7	35.7
Minimum temperature of the coldest month (BIO6, C°)	6.6	4.3	9.8	1.2	6.0	6.4	7.1	0.7	1.6
Temperature Annual Range (BIO7 = BIO5-BIO6, °C)	22.5	18.1	25.2	1.8	21.4	22.7	23.7	40.0	39.9
Average temperature of the wettest quarter (BIO8, °C)	12.8	10.8	14.2	1.0	12.1	12.5	13.9	16.2	14.6
Average temperature of the driest quarter (BIO9, °C)	24.0	23.3	24.5	0.4	23.6	23.9	24.4	21.8	22.33
Average temperature of the warmest quarter (BIO10, °C)	24.3	23.7	24.8	0.4	24.0	24.3	24.6	24.4	25.3
Average temperature of the coldest quarter (BIO11, °C)	12.4	10.8	13.2	0.6	12.1	12.5	12.7	11.8	12.8
Annual precipitation (BIO12, mm)	775.8	661.0	873.0	54.2	750.0	779.0	811.0	563.4	736.8
Precipitation of wettest month (BIO13, mm)	146.6	123.0	167.0	12.9	138.0	143.0	161.0	152.0	232.8
Precipitation of driest month (BIO14, mm)	0.4	0.0	2.0	0.6	0.0	0.0	1.0	0.0	0.0
Precipitation seasonality (BIO15, %)	81.8	74.1	87.3	4.6	76.9	82.8	85.8	117.8	106.3
Precipitation of wettest quarter (BIO16, mm)	398.7	334.0	468.0	36.7	377.0	396.0	429.0	129.8	141.4
Precipitation of driest quarter (BIO17, mm)	24.0	23.3	24.5	0.4	23.6	23.9	24.4	0.1	1.5
Precipitation of warmest quarter (BIO18, mm)	24.3	23.7	24.8	0.4	24.0	24.3	24.6	1.3	4.5
Precipitation of coldest quarter (BIO19, mm)	12.4	10.8	13.2	0.6	12.1	12.5	12.7	44.9	51.6

### Agro-morphological characterization

The results revealed that all the agro-morphological parameters of *S. flexuosa* (L.) Medik. were significantly (*p* < 0.01) influenced by the ecotype and phenological stage (Table 2), whereas the year significantly (*p* < 0.05) influenced all the parameters except DMY. The interactions ecotype × year and ecotype × phenological stage showed significant interaction (*p* < 0.01) for all the agronomical and morphological parameters. The interaction year × phenological stage had a highly significant influence (*p* < 0.001) on the number of plagiotropic branches, leaves, and inflorescences per plant and the length of the longest plagiotropic branch per plant. The interaction ecotype × year × phenological stage was significant (*p* < 0.05) for all parameters except DMY. Several authors reported climatic conditions^40^ effects on plant development, which could explain the ecotype × year interactions for all the morphological parameters, as the 2020 growing season was 31% wetter than one of 2019.

**Table 2:** Descriptive statistics and means of agro-morphological traits of 21 cultivated *S. flexuosa* (L.) Medik. ecotypes across phenological stages [*B* Budding, *SF* Start of flowering and *FF* Full flowering] and years (2019 and 2020).

	Mean	SEM	Min.	Max.	CV (%)	Phenological stage (PS)			Year (Y)		Ecotype (E)	Y	PS	E × Y	E × PS	Y × PS	E × Y × PS
						B	SF	FF	2019	2020							
Number of plagiotropic branches	9.1	0.1	4.9	14.9	15.4		9.0	9.3	8.5	9.8	***	***	**	***	**	***	***
Plant height (cm)	65.2	0.8	40.7	107.4	16.6		61.7	68.8	64.0	66.5	***	**	***	***	***	ns	***
Length of the orthotropic axis (cm)	60.5	0.7	36.4	98.7	18.1		57.0	64.0	59.8	61.2	***	**	***	***	***	ns	***
Length of the longest plagiotropic branch (cm)	106.8	1.3	66.8	138.3	19.0		99.2	114.4	92.4	121.2	***	***	***	***	***	ns	***
Number of leaves per plant	344.8	8.8	164.0	861.2	24.7		326.9	362.7	261.9	427.6	***	***	***	***	***	***	***
Number of inflorescences per plant	159.8	3.9	79.9	316.6	35.9		143.4	176.1	148.7	170.9	***	***	***	***	***	***	***
Number of total branches	164.5	3.6	70.2	413.6	24.2		159.5	169.4	152.9	176.0	***	***	**	***	***	ns	***
Stem diameter (cm)	1.1	0.0	0.9	1.5	12.6		1.1	1.1	1.1	1.1	***	*	***	***	***	ns	***
Fresh matter yield (T/ha)	33.0	0.6	19.6	50.0	3.8	28.8^c^	32.9^b^	37.2^a^	32.6	33.3	***	***	***	***	***	ns	***
Dry matter yield (T/ha)	4.8	0.1	3.0	7.2	5.8	3.8^c^	4.8^b^	5.9^a^	4.8	4.9	***	ns	***	***	***	ns	ns
Leaf-to-stem ratio	0.57	0.01	0.39	0.84	6.00	0.69^a^	0.56^b^	0.47^c^	0.55	0.59	***	***	***	***	***	ns	*
Number of days to emergence (°C)	89.3	1.8	64.6	115.0	6.8						***						
Flower bud appearance (°C)	525.3	4.0	473.3	587.9	1.2						***						
Start of flowering (°C)	613.2	3.5	540.2	689.8	0.4						***						
Full flowering (°C)	642.8	2.7	605.0	703.4	1.4						***						
End of flowering (°C)	758.1	6.3	695.8	831.5	5.2						***						
Flowering duration (°C)	146.9	4.1	98.7	241.7	5.2						***						
Thousand seeds weight (g)	5.9	0.1	4.6	6.9	2.8						***						
Seed weight per plant (g)	23.6	0.5	17.1	31.0	4.9						***						

*SEM* Standard error of the mean, *Min.* Minimum, *Max.* Maximum, *CV* Coefficient of variation (%). ns, * , ** and *** represent non-significant, significant at *p* < 0.05, *p* < 0.01, and *p* < 0.001, respectively, ^a,b,c^ data within a row with different superscripts are significantly different (*p* < 0.05).

The number of plagiotropic branches varied greatly between ecotypes, but the average number (9.1) was in the range of values reported for alfalfa (*Medicago sativa*)^41^, but higher than values reported for *S. coronaria* (L.) Medik. (4.4)^5^. The same observation was made for the number of total branches, with an average of 165 in the present trial versus 43 for *S. coronaria* (L.) Medik.^﻿5^. The increase in the number of plagiotropic (9.8 vs. 8.5) and total branches (176 vs. 153) in the second year was probably due to the higher water and light availability than in the first year. Indeed, in a wet year, more water is available for plant growth, which can lead to increased vegetative growth and branching. Also, plagiotropic branches, which grow horizontally, could enhance light capture efficiency and increase the amount of light received by the plant. In a wet year, there could be more diffuse light due to cloud cover, which can also promote branching^42^.

The average plant height of *S. flexuosa* (L.) Medik. was 65 cm. Values of 40 cm and 60 cm have been reported for *S. coronaria* (L.) Medik. and alfalfa, respectively^5,41^. The length of the longest plagiotropic branch per plant was about 107 cm compared to 159 cm observed for *S. coronaria * (L.) Medik.﻿﻿^43^. Thus, this last one tends to grow horizontally compared to *S. flexuosa* (L.) Medik., which would grow more vertically. The average main stem diameter (1.07cm) of the ecotypes in this study was smaller than the average diameter observed for *S. coronaria* (L.) Medik. ﻿in Spain (1.9 cm)^9^. This smaller stem diameter with a higher height of *S. flexuosa* (L.) Medik. could make it more susceptible to lodging.

The wide variability of morphological parameters (with 12.6% for stem diameter and 35.9% for the number of inflorescences per plant) between ecotypes reflected the low level of human intervention for selection. The low variability for phenology (with 0.2% for the start of flowering and 6.8% for the number of days to emergence) was also reported for other *legume* species^44^. Concerning yield parameters, *S. coronaria* (L.) Medik.﻿ is a biennial fodder, while *S. flexuosa* (L.) Medik. is annual^5,16^. However, the annual observed fresh matter yield (33.0 T/ha) in the present study was close to that reported by Córdoba et al.^9^ (29.0 T/ha) with biennial populations of *S. coronaria* (L.) Medik.﻿ in the first year.

In contrast, DMY was higher in the present trial (4.8 vs. 3.5 T DM/ha). This difference in DM content could be explained by the lower leaf-to-stem ratio (0.55) due to greater branching in *S. flexuosa* (L.) Medik. compared to the one of *S. coronaria* (L.) Medik. ﻿(0.4 to 3.2)^5,45^. Moreover, the *S. flexuosa* (L.) Medik plants should be handled with care as wild species are reported to be less resilient compared to crop progenitors in their response to defoliation^46^. This results from selecting traits that allow crops to recover quickly from damage caused by grazing or harvesting.

In this study, all the ecotypes in 2020 were leafier and denser than in 2019 (428 vs. 262 for the number of inflorescences per plant and 176 vs. 153 for the number of total branches). It could be due to higher rainfall during the second growing season (736.8 vs. 563.2 mm). However, only an increase in fresh matter yield and no significant increase in DM yield were observed. It was possibly explained by the increase in leaf-to-stem ratio as DM of leaves is lower compared to the stems.

Concerning seed yield parameters, thousand seed weight averaged 5.9 g and appeared in the range of values reported for *S. coronaria* (L.) Medik.^﻿9^. Those values are interesting as crop species are reported to have a higher thousand seed weight than wild species due to artificial selection for larger seeds, which can improve competitive ability during the early stages of plant establishment and increase crop yield and growth^46^.

The phenology and optimal conditions for each phase of the crop cycle are essential in deciding the most appropriate ecotypes for a particular region. Even under favorable conditions (greenhouse sowing in trays), the number of days between sowing and emergence of *S. flexuosa* (L.) Medik. seeds, calculated in GDD, varied from 64.6 to 115.0 °C, with an average of 89.3 °C, showing a powerful impact of the ecotype on this parameter. The appearance of the first flower bud (525.3 °C) and the early (613.2 °C), full (642.8 °C), and late (758.1 °C) stages of flowering were also significantly (*p* < 0.001) different between ecotypes.

### Nutritional characterization

All the nutritional parameters (Table 3) of *S. flexuosa* (L.) Medik. were significantly influenced (*p* < 0.001) by the ecotype and the phenological stage. Likewise, the year effect had a significant (*p* < 0.05) influence on all nutritional parameters except on Ash, IVECPD, and ME. The ecotype × year interaction effect was highly significant (*p* < 0.001) for all parameters, possibly due to the differences in the environmental conditions, especially in the precipitations between years, or in the genetics that affect their response to environmental variation. The ecotype × phenological stage interaction effect was highly significant (*p* < 0.01) for all parameters except for Ash and ADF. The year × phenological stage interaction effect was significant (*p* < 0.05) for all parameters except for DM, Ash, CF, ADL, and IVTD. Finally, the triple interaction was significant (*p* < 0.05) for Fat, ADF, IVTD, Phenols, NTP, CT, and HT. It could be explained that for each phenological stage, it was not the same ecotypes that were the more performant. Thus, the accurate phenological stage must be determined for each ecotype to ensure its best results.

**Table 3:** Descriptive statistics and means of nutritional traits of 21 cultivated Moroccan *S. flexuosa* (L.) Medik. ecotypes across phenological stages [*B* Budding, *SF* Start of flowering and *FF* Full flowering] and years (2019 and 2020).

	Mean	SEM	Min.	Max.	CV (%)	Phenological stage (PS)			Year (Y)		Ecotype (E)	Y	PS	E × Y	E × PS	Y × PS	E × Y × PS
						B	SF	FF	2019	2020							
Dry matter (DM, %)	14.55	0.11	12.48	16.59	5.12	13.27^c^	14.56^b^	15.82^a^	14.80	14.29	***	***	***	***	**	ns	ns
Crude proteins (% DM)	19.43	0.16	17.80	21.52	4.53	21.79^a^	19.82^b^	16.99^c^	19.31	19.76	***	***	***	***	***	***	ns
Crude protein yield (T/ha)	0.93	0.02	0.56	1.51	6.25	0.83^c^	0.95^b^	1.00^a^	0.92	0.93	***	*	***	***	***	*	ns
Fat (% DM)	2.49	0.03	2.04	2.98	6.23	2.22^c^	2.52^b^	2.72^a^	2.46	2.52	***	**	***	***	***	***	***
Ash (% DM)	14.09	0.10	12.22	16.42	4.90	12.99^c^	14.66^b^	14.81^a^	14.17	14.01	***	ns	***	***	ns	ns	ns
Crude fiber (% DM)	24.35	0.35	20.16	32.28	5.65	24.11^b^	19.58^c^	29.38^a^	23.99	24.72	***	***	***	***	***	ns	ns
Neutral detergent fiber (% DM)	47.12	0.21	44.68	52.03	1.80	44.57^c^	46.92^b^	49.87^a^	48.12	46.12	***	***	***	***	***	**	ns
Acid detergent fiber (% DM)	32.20	0.18	29.64	34.76	5.04	32.05^b^	29.79^c^	34.59^a^	32.31	32.10	***	***	***	***	***	***	*
Acid detergent lignin (% DM)	14.12	0.15	11.03	18.00	3.24	12.79^c^	14.23^b^	15.35^a^	14.25	13.99	***	***	***	***	ns	ns	ns
Nitrogen-free extract (% DM)	39.54	0.34	31.52	45.41	4.34	38.89^b^	43.63^a^	36.10^c^	40.08	39.00	***	**	***	***	***	ns	ns
In vitro enzymatic crude proteins digestibility (% DM)	43.68	0.15	40.91	49.53	2.25	43.69^b^	44.87^a^	42.46^c^	43.68	43.68	***	ns	***	***	***	*	ns
In vitro enzymatic organic matter digestibility (% DM)	61.36	0.22	58.30	65.02	1.64	64.58^a^	61.25^b^	58.25^c^	61.12	61.60	***	***	***	***	***	*	ns
In vitro true digestibility (% DM)	68.45	0.34	61.85	73.33	1.72	73.07^a^	68.33^b^	63.95^c^	68.22	68.68	***	**	***	***	***	ns	***
Metabolizable energy (MJ/kg DM)	8.88	0.15	8.16	9.72	4.39	9.82^a^	8.90^b^	7.92^c^	8.87	8.89	***	ns	***	***	***	***	ns
Phenols (% DM)	3.62	0.08	1.99	5.43	2.26	4.50^a^	3.55^b^	2.82^c^	3.68	3.57	***	***	***	***	***	***	***
Non-tannic phenols (% DM)	1.72	0.04	0.72	2.97	5.54	2.16^a^	1.66^b^	1.34^c^	1.75	1.70	***	***	***	***	***	***	***
Condensed tannins (% DM)	1.11	0.03	0.68	1.89	5.85	1.37^a^	1.07^b^	0.88^c^	1.16	1.05	***	***	***	***	***	***	***
Hydrolysable tannins (% DM)	0.79	0.06	0.40	1.58	17.24	0.96^a^	0.82^b^	0.60^c^	0.77	0.81	***	**	***	***	***	*	***

*SEM* Standard error of the mean, *Min.* Minimum, *Max.* Maximum, *CV* Coefficient of variation (%). ns, *, ** and *** represent non-significant, significant at *p* < 0.05, *p* < 0.01, and *p* < 0.001, respectively, ^a,b,c^ data within a row with different superscripts are significantly different (*p* < 0.05).

Legumes are considered a supplementary high-quality protein-based feed. Nutritional values can vary among species and cultivars, throughout the years and between phenological stages^9,10,47^. *Sulla flexuosa* (L.) Medik. showed a high average CP content (19.4% DM) which is close to values reported by Zirmi-Zembri et al.^48^ for *S. flexuosa* (L.) Medik. and values reported by Borreani et al.^10^ for *S. coronaria* (L.) Medik. All the ecotypes had CP content higher than 9%, the minimum level required for adequate microbial synthesis in the rumen^49^. Both NDF (47.1 vs. 48.6% DM) and ADF (32.2 vs. 34.5% DM) contents were also close to values observed by Kadi et al.^50^, while for ADL, the values were almost two times higher (14.1 vs. 9.0% DM), respectively.

With the advancement of the phenological stage of the plants (Table 3), a significant (*p* < 0.001) increase in Fat (+ 22.5%), Ash (+ 14.0%), CF (+ 21.8%), NDF (+ 11.9%), ADF (+ 7.9%) and ADL (+ 20.0%) contents as well as an equally significant decrease in NFE (−7.2%), in ME (−19.3%) and in the OM (−9.8%), crude protein (−2,8%), and true (−12.5%) digestibilities were observed. The start of flowering showed lower CF and ADF contents than the other two stages (budding and full flowering). Increasing fresh or dry matter yield usually leads to higher contents of NDF because of increased stem proportion, cell wall thickening, or lower protein and other soluble cell contents^51^. These changes in plant development occur with the advancing of the phenological stage^10^. Phenols, NTP, CT, and HT decreased by an average of 37% from the budding to the full flowering stage. The results of the present study concerning CT were lower than the ranges obtained for *S. coronaria﻿* (L.) Medik.^11^. This decrease in the present trial could be due to decreased leaf-to-stem ratio, as condensed tannins are concentrated in leaves for the close species *S. coronaria* (L.) Medik.﻿^11^.

As expected, CP decreased by 22% DM from the budding to the full flowering stage. It could be due to a growth dilution effect due to increased structural carbohydrates with the advancing maturity stage. However, although the CP percentage decreased, the crude protein yield increased significantly (*P* < 0.001) with the phenological stage and, thus, with the increasing yield.

*Sulla flexuosa* (L.) Medik. ecotypes showed little increase in CP (+ 2.3%), Fat (+ 2.4%), and CF (+ 3.0) contents, and a small decrease in NDF (−4.2%), ADF (−0.7%), and ADL (−1.8%), NFE (−2.7%) contents between the two years. Slow increases (< 1.0%) were found for OM and true digestibilities. Phenols, NTP, and CT were significantly (*P* < 0.05) 3.0%, 2.9%, and 9.5% lower in the second year, respectively. However, HT contents were 5.2% higher. The low increase in fibers during the second year could be due to low temperature differences between the two years because plant development and fiber accumulation were reported to be negatively related to temperature^52^. It was stated that higher temperatures of about 1.5 °C in crop season led to 3.5% higher NDF and 21% higher ADL contents. However, in the present study, it was only a 0.4 °C increase. The absence of differences between the overall growth, shown by the same yields between the two years, resulted in a weak change in fiber accumulation^53^. The CP Digestibility (43.7%) was close to values obtained for lucerne (46.4% DM)^54^ and was not influenced by the year. The differences observed with the CP digestibility could be explained by the fiber contents and the inverse relationship between these two parameters. The increased fiber content with the phenological stage also explained the decreased ME. However, ME always ranged in values reported for sainfoin and alfalfa in Turkey^42^.

### Correlation matrix

#### Correlation between morpho-pheno-agromorphological and edapho-climatic parameters

**Figure 2:**
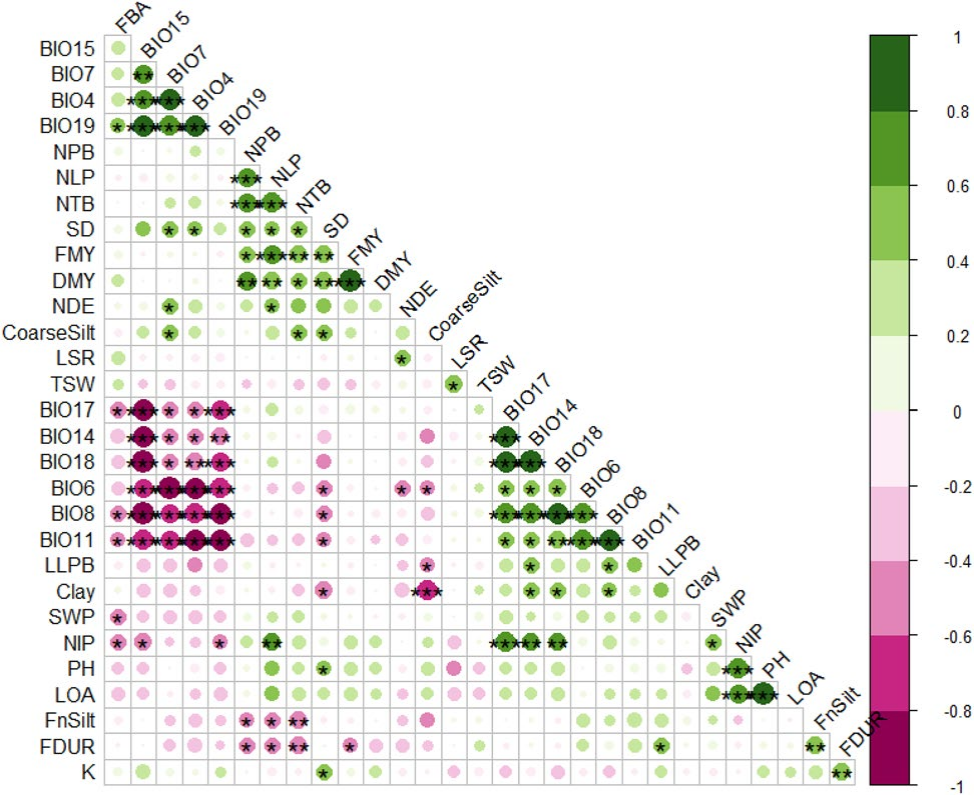
Correlation matrix of agro-morphological and edapho-climatic traits evaluated in 21 Moroccan *S. flexuosa* (L.) Medik. ecotypes. Positive correlations are displayed in green and negative correlations in purple color. The color intensity and the size of the circle are proportional to the correlation coefficients. On the right side of the correlogram, the legend color shows the correlation coefficients and the corresponding colors. *NPB* Number of plagiotropic branches, *PH* Plant height (cm), *LOA* Length of the orthotropic axis (cm), *LLPB* Length of the longest plagiotropic branch (cm), *NLP* Number of leaves per plant, *NIP* Number of inflorescences per plant, *NTB* Number of total branches, *SD* Stem diameter (cm), *FMY* Fresh matter yield (T/ha), *DMY* Dry matter yield (T/ha), *LSR* Leaf to stem ratio, *NDE* Number of days to emergence (°C), *FBA* First bud appearance (°C), *FDUR* Flowering duration (°C), *SWP* Seed weight per plant (g), *K* Exchangeable potassium (ppm), *FnSilt* Fine silt (%), *CoSilt* Coarse silt (%), *BIO4* Temperature seasonality (standard deviation × 100), *BIO6* Minimum temperature of the coldest month (C°), *BIO7* Average temperature of the wettest quarter (°C), *BIO8* Average temperature of the wettest quarter (C°), *BIO11* Average temperature of the coldest quarter (°C), *BIO15* Precipitation seasonality (coefficient of variation, %), *BIO18* Precipitation of warmest quarter (mm), *BIO19* Precipitation of coldest quarter (mm).

Understanding the correlations between different parameters is crucial because it allows the accomplishment of the indirect selection of the parameters that are inherited quantitatively and influenced by genetic effects^55^. The Pearson correlation coefficient between pairs of the parameters and the associated probabilities is given in Fig. 2.

As expected, some agro-morphological parameters were correlated. The number of days to emergence was negatively correlated to the minimum temperature of the coldest month. It could be an adaptation strategy of ecotypes to cold winter. Annicchiarico et al.^5^ found that ecotype adaptation to cold winter was associated with latitude and, more specifically, the extent of cold stress in collecting sites. However, the appearance of the first flower bud was positively correlated to the mean temperature of the wettest quarter, the mean temperature of the coldest quarter, and the precipitation of the driest quarter, which confirms the results of Iannucci et al.^56^ for Mediterranean species, including *S. coronaria* (L.) Medik. ﻿that was strongly influenced by increasing temperature in accelerating the development rate and in earliness for time to flowering.

The appearance of the first flower bud was also positively correlated to precipitation of the coldest quarter, which confirms the relationship between precipitation and phenology. A plant could adjust its maturity to soil moisture availability to facilitate seedset before the start of the dry summers faced in the Mediterranean region^57^. Accordingly, the negative correlation between the number of inflorescences per plant and the first bud appearance exhibits the effect of dry conditions on the reproductive parameters of the plants^58^.

Issolah and Khalfallah^59^ stated that populations of *S. coronaria* (L.) Medik.﻿ with high thousand seeds weight are native to high altitude regions, while no significant correlation was reported for *S. flexuosa* (L.) Medik. ecotypes, probably due to low ranges of altitudes of collection sites (9–350 m) compared to *S. coronaria* (L.) Medik.,﻿ which can be found at more than 1000 m^47^. This study confirms previous studies of annual legumes by Pecetti et al.^57^ and Berger et al.^60^ that natural selection for flowering time is strongly influenced by eco-geographic variables (precipitations and temperature).

This study showed that ecotypes with a high number of plagiotropic branches had highly branched axes, which agrees with a study on *S. coronaria* (L.) Medik.^﻿61^. In the present study, high total branches were also correlated to dry matter yield, as reported by a previous study^53^. Increasing *S. flexuosa* (L.) Medik. DM yield is an important goal in future breeding. This study showed significant positive correlations between DM yield and the number of plagiotropic branches, total branches, and leaves per plant, which agrees with previous studies on other legumes^53^. The present results also indicated that selection for high DM yield of *S. flexuosa* (L.) Medik. could be accomplished by selecting dense and leafy plants. All these characteristics could contribute to increased photosynthetic activity, leading to higher DM production. However, the leafy nature might be a disadvantage in dry areas as it facilitates rapid water loss through transpiration^62^. In the case of other legumes, the longer stems length is translated into a higher total DMY^53^. However, no significant correlation was observed between those parameters in the present study. As mentioned earlier, these results undoubtedly depended on the plant genetic background and the environmental and growth conditions. Indeed, in the present study, we revealed the geographical and bioclimatic origins of *S. flexuosa* (L.) Medik. ecotypes were important in plant adaptation due to their correlations with reproductive and phenological parameters.

#### Correlation between nutritional and edapho-climatic parameters

**Figure 3:**
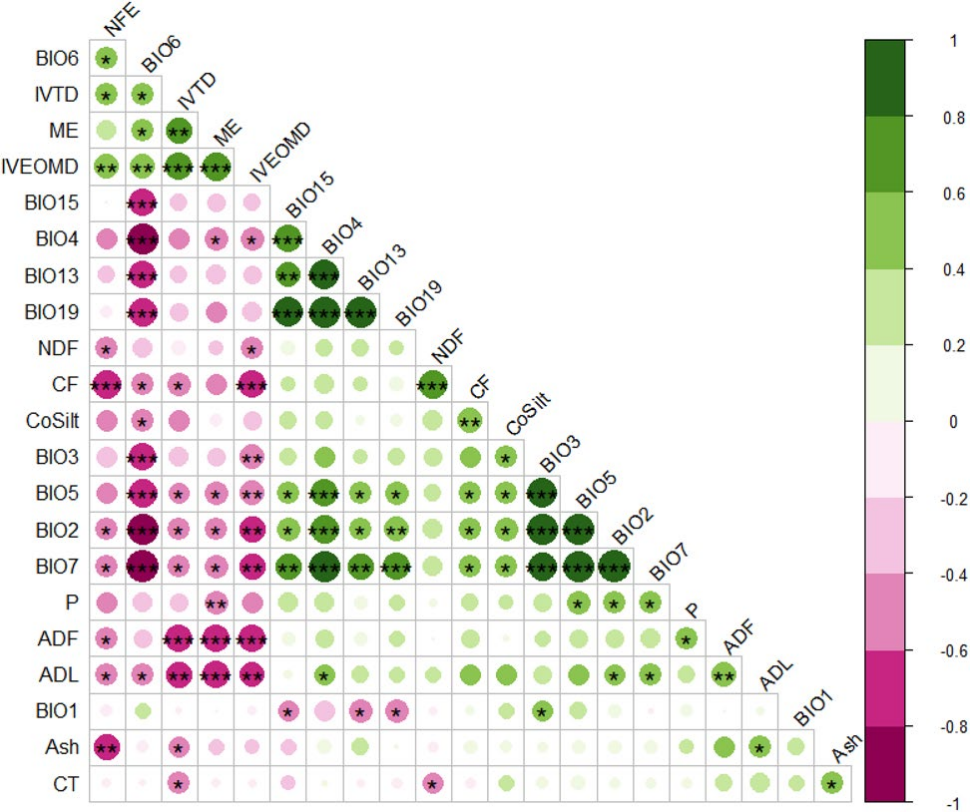
The correlation matrix of nutritional and edapho-climatic traits evaluated in 21 Moroccan *S. flexuosa* (L.) Medik. ecotypes. Positive correlations are displayed in green and negative correlations in purple color. The color intensity and the size of the circle are proportional to the correlation coefficients. On the right side of the correlogram, the legend color shows the correlation coefficients and the corresponding colors. Ash (% DM), *NFE* Nitrogen-free extract (% DM), *CF* Crude fibers (% DM), *NDF* Neutral detergent fibers (% DM), *ADF* Acid detergent fibers (% DM), *ADL* Acid detergent lignin (% DM), IV*EOMD* Enzymatic organic matter digestibility (%), *IVTD* In vitro true digestibility (%), *ME* Metabolizable energy (MJ/kg DM), *CT* Condensed tannins (% DM), *CoSilt* Coarse silt (%), *P* Available phosphorus (ppm), *BIO1* Average annual temperature (°C), *BIO2* Average diurnal variation [monthly average (max temperature—min temperature)] (°C), *BIO3* Isothermality (BIO2/BIO7 × 100)], *BIO4* Temperature seasonality (standard deviation * 100), *BIO5* Maximum temperature of the warmest month (C°), *BIO7* Average temperature of the wettest quarter (°C), *BIO13* Precipitation of wettest month (mm), *BIO15* Precipitation seasonality (coefficient of variation), *BIO19* Precipitation of coldest quarter (mm).

The second hypothesis was that variation in nutritional parameters was related to the eco-geography of their collection sites (Fig. 3). No significant correlation was found between DM yield and whole plant CT content, suggesting that selection must be based on the dry matter yield of ecotypes and the targeted CT content. The leaf-to-stem ratio is considered a positive indicator of forage quality due to its close association with forage digestibility and intake^10^. Paradoxically, in this study, there was no significant correlation between the leaf-to-stem ratio and the digestibilities or the different fiber contents. Links between higher temperatures during the growing season and declining nutritional values have been established under controlled conditions^63^. The negative correlation of ADL and CF with the minimum temperature of the coldest month (BIO6) was similar to results found by Ruisi et al.^47^ for *S. coronaria* (L.) Medik. They reported that populations from a higher altitude with higher rainfall and lower temperature were characterized by small leaves and plants and low total biomass production. In the present study, fresh matter and DM yields were positively correlated to the number of total branches, which were richer in fibers than leaves. The positive correlation of ADL and CF with average diurnal variation (BIO2) and annual temperature range (BIO7) could be due to rising temperatures as forage quality declines with rising temperatures^63^. The same explanation also goes for NFE as it was positively correlated to OM and true digestibilities, and negatively to BIO2, BIO7, ADL, and CF. High long-term phosphorus fertilization was reported to increase ADF accumulation in alfalfa^64^. In the present study, the positive correlation between ADF and P could be explained by the fact that ecotypes adapted to low P amounts in their collection sites could not use the P present in the experimental field due to fertilization and thus had a lower fiber production. The true digestibility was higher than OM digestibility. It could be due to the methodology where the true digestibility included rumen flora, which increased digestibility compared to enzymatic digestibility, which used commercial enzymes.

### Principal component analysis (PCA)

**Figure 4:**
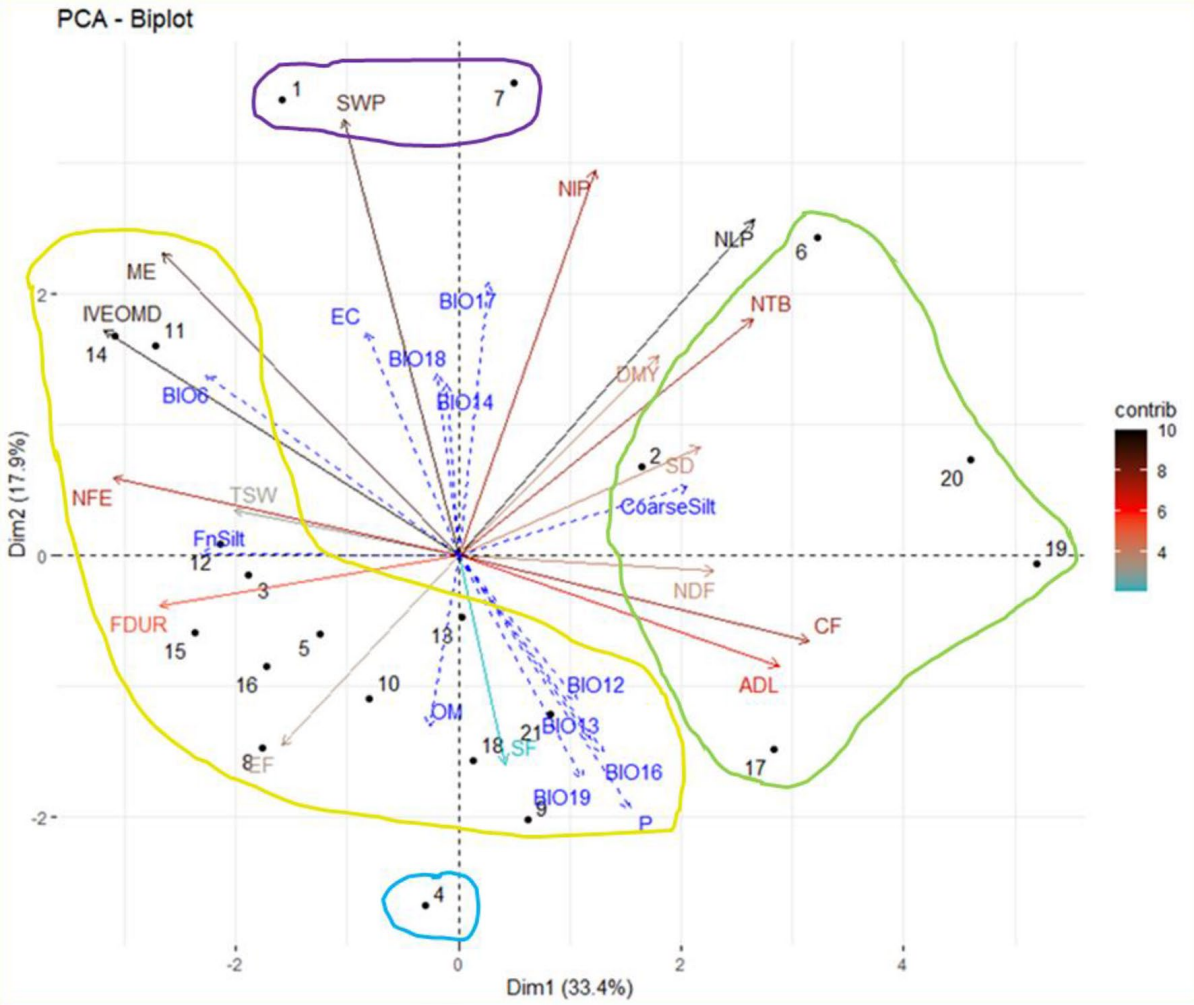
Graph of the variables and individuals of the principal component analysis. *NLP* Number of leaves per plant, *SWP* Seeds weight per plant (g), *TSW* Thousand seed weight (g), *NIP* Number of inflorescences per plant, *NTB* Number of total branches, *SD* Stem diameter (cm), *DMY* Dry matter yield (T/ha), *CF* Crude fiber (% DM), *NDF* Neutral detergent fibers (% DM), *ADF* Acid detergent fibers (% DM), *ADL* Acid detergent lignin (% DM), *IVEOMD* Enzymatic organic matter digestibility (%), *NFE* Nitrogen-free extract (% DM), *ME* metabolizable energy (MJ/kg DM), *Hum.* Humidity (%), *FnSilt* Fine silt (%), *P Available* phosphorus (ppm), *EC* Electrical conductivity (mS/m), *OM* Organic matter (%), *BIO6* Min temperature of coldest month (°C), *BIO12* Annual precipitation (mm), *BIO13* Precipitation of wettest month (mm), *BIO14* Precipitation of driest month (mm), *BIO16* Precipitation of wettest quarter (mm), *BIO17* Precipitation of driest quarter (mm), *BIO18* Precipitation of warmest quarter (mm), *BIO19* Precipitation of coldest quarter (mm). Four clusters were determined by the cluster heatmap analysis and represented via the four colored circles (green, purple, yellow, and blue) on this figure.

The PCA is an important tool in determining the most important variables contributing to variation^65^. The PCA for quantitative parameters (Fig. 4) showed that the major part of variation (64.9%) in *S. flexuosa* (L.) Medik. ecotypes was explained by the first three components. The PC1, the most important component, explained 33.4% of the total variation with the positive contribution of CF, ADL, SD, NDF, and DMY and the negative contribution of OM Digestibility, ME, NFE, and thousand seed weight. The PC1 was positively correlated to the contents of different fibers and negatively to digestibility. A low nutritional value, therefore, determined the PC1 axis. The second component axis accounted for 17.9% of the total variation, and the traits with a positive weight on this component were the seed weight per plant and the number of inflorescences per plant, while the start and the end of flowering had a negative weight. Plant reproductive parameters and phenology, therefore, determined the PC2 axis. The edapho-climatic parameters (Table 1) were used as supplementary variables. The annual precipitation (BIO12), the precipitation of the wettest month (BIO13), the precipitation of the wettest quarter (BIO16), and the precipitation of the coldest quarter (BIO19), which are indicative of an abundance of precipitations were at the same direction of phenology parameters (start and end flowering). In the Mediterranean area, several authors reported late flowering for ecotypes that have sufficient water supply to take advantage of the long season for a high yield and not overlap the harvesting season with rainfall during haymaking^47^. However, the precipitation of the driest month (BIO14), the precipitation of the driest quarter (BIO17), and the precipitation of the warmest quarter (BIO18) were in the opposite way of the phenology parameters, showing that also extreme drought leads to late flowering. Annicchiarico et al.^5^ also reported low adaptation of *S. coronaria* (L.) Medik. ﻿to drought summer compared to winter cold. The minimum temperature of the coldest month (BIO6), indicative of extreme coldness, was in the same direction as nutritional value parameters (ME, OM Digestibility, and NFE). It could be due to the sensitivity of plants to cold, which leads to small developed plants with low branches and fibers and thus with high digestibility^5^.

### Heatmap analysis

**Figure 5:**
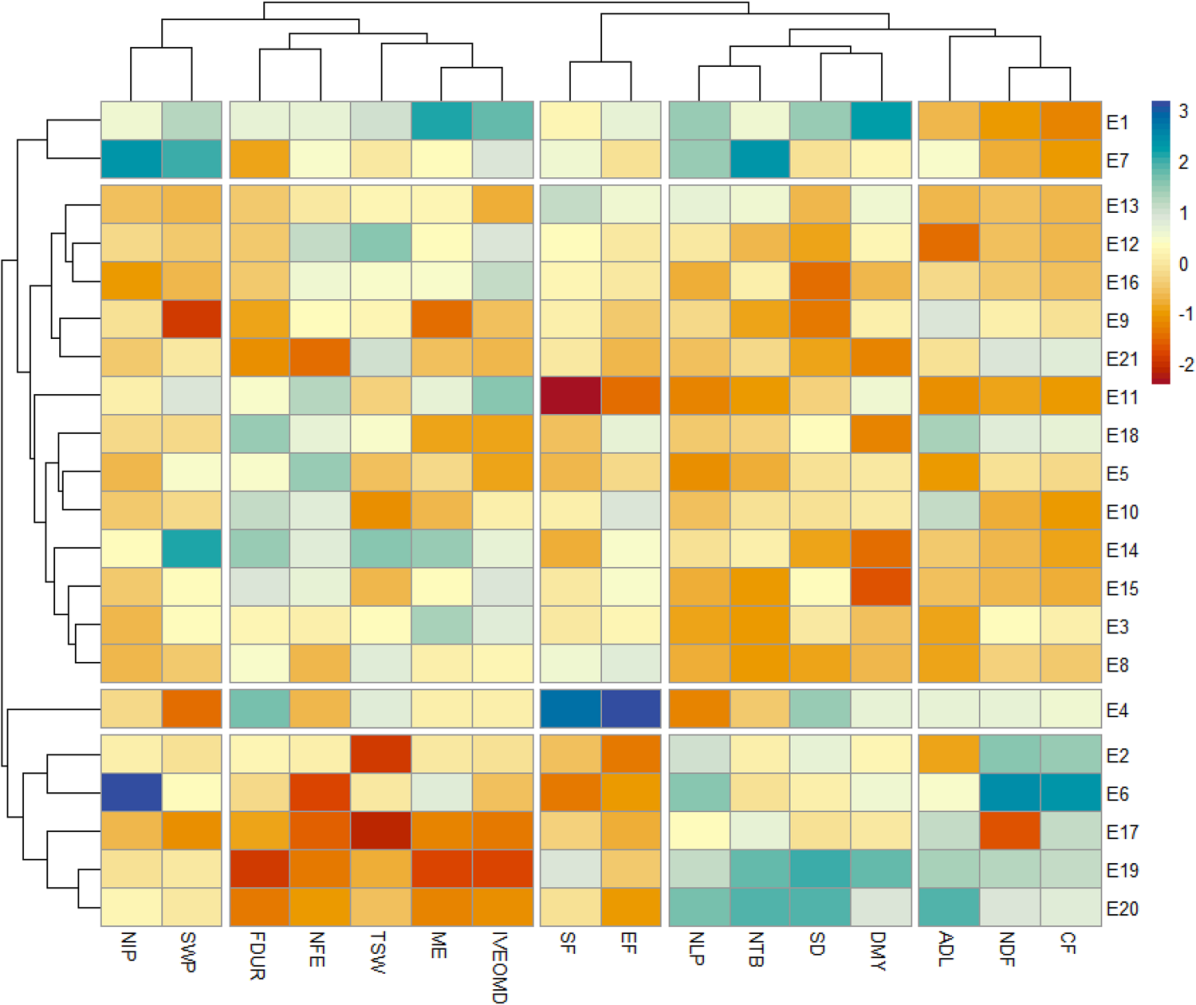
Cluster heatmap analysis of *S. flexuosa* (L.) Medik. ecotypes’ responses to morpho-phenological, agronomic, and nutritional characterization. The heatmap plot describes the relative abundance of each *S. flexuosa* (L.) Medik. ecotype (rows) within each trait (column). The color code (blue to dark red) displays the values of the parameters: blue color indicates high values while red color indicates low values. The dendrogram (on the left) shows the hierarchical clustering of *S. flexuosa* (L.) Medik. ecotypes based on the Euclidian distance and Ward’s clustering method. *CF* Crude fibers (% DM), *NDF* Neutral detergent fibers (% DM), *ADL* Acid detergent lignin (% DM), *DMY* Dry matter yield (T/ha), *SD* Stem diameter (cm), *NTB* Number of total branches, *NLP* Number of leaves per plant, *EF* End of flowering (°C), *SF* Start of flowering (°C), *IVEOMD* Enzymatic organic matter digestibility, *ME* Metabolizable energy (MJ/kg DM), *TSW* Thousand seed weight (g), *NFE* Nitrogen-free extract (% DM), *FDUR* Flowering duration (°C), *SWP* Seed weight per plant (g), *NIP* Number of inflorescences per plant.

A heatmap analysis was performed to visualize and investigate more detailed differences between the ecotypes based on morphological, agronomic, and nutritional parameters. In contrast to clustering methods, which allow the clustering of ecotypes on groups with similar characteristics, heatmap identifies differences at the cluster’s level. The heatmap analysis showed a couple of dendrograms. The first one (dendrogram1) structured on the left, an arrangement that corresponds to the *S. flexuosa* (L.) Medik. ecotypes, and the second one, on the top (Dendrogram2), clustered the agro-, morpho-, pheno-, and nutritional parameters that affected the dendrogram 1 distribution. These parameters were classified into five groups of fibers (CF, NDF, and ADL), fodder production (DMY, stem diameter, number of total branches, and number of leaves per plant), phenology (start and end of flowering), reproductive parameters (seed weight per plant and number of inflorescences per plant), and nutritional value (OM Digestibility, ME and thousand seed weight, NFE and flowering duration). The heatmap figure (Fig. 5) displayed four main groups of ecotypes: the first one above, which corresponds to E1 and E7, E4 was clustered as the third group, E2, E6, E17, E19, and E20 that formed the fourth group and the second group with the rest of the ecotypes.

Low fiber values characterized the first and second groups, while the third and fourth groups were characterized by the opposite. Contrary to the other groups, lower yield parameters characterized the second group. The third and fourth groups were opposite according to phenology. The first and second groups were intermediate. The fourth group was characterized by low nutritional value. Except for the first group, the other groups had low reproductive parameters. In the present collection, extreme ecotypes are found. For example, in some studies, DMY was positively correlated to NDF and ADF^53^. However, the E1 was the best ecotype because it had the highest productivity and nutritional value (low NDF and CF, high DMY, ME, and OM digestibility), with a high stem diameter, which means it is also protected from lodging^66^. This ecotype was of dual purpose because it also showed high reproductive parameters, which are important in seed dissemination. High thousand seed weight is related to the high germination of these seeds^67^. At a very long phenotypical distance, E21 is the opposite regarding low DMY and NFE and intermediate fibers (NDF, ADF, ADL, CF).

E11 could also be interesting related to its precocity at the start and end of flowering and its long flowering duration, which is reflected by a high seed weight per plant. E4 is very late (high start and end of flowering). The literature shows that ecotypes with long vegetative growth tend to have high DMY^47^. However, E4 showed intermediate DMY, probably due to its low number of leaves per plant. Porqueddu et al.^68^ stated that nutritional value in legumes tends to be higher in late than in early maturing cultivars. Indeed, the early maturing cultivars start to bloom earlier and have a higher proportion of stems, stem proportion being negatively correlated to digestibility. Ecotype 4 could be interesting for breeding because it was the late ecotype, even though it showed chemical composition and nutritional value close to the mean of all ecotypes. The choice of an early or late ecotype will depend on the climatic conditions in which they are seeded. In less humid conditions, an early ecotype will take advantage of the early rains to develop rapidly. In wetter conditions, the development of a late ecotype will allow a cut during a drier period, thus reducing the risk of rain during the drying process in the field in haymaking. Since there is no correlation with forage yield, choosing ecotypes that are early or late and very productive will be necessary. However, if the aim is seed dissemination to conserve *S. flexuosa* (L.) Medik. in pastures, early ecotypes will be preferred to avoid water stress during seed maturation.

The clustering of these ecotypes had the major objective of creating a *S. flexuosa* (L.) Medik variety. Ceccarelli and Grando^69^ stated that during selection, despite that the participatory variety selection is technically easier to organize due to the limited number of lines that usually reach the final stage of selection, it is important to use the participatory plant breeding and involve farmers, from the beginning, in most important decisions, during all the stages of a plant breeding program. Otherwise, there is a risk of discarding potentially desirable breeding material to farmers. By involving farmers in the selection process, participatory plant breeding can also help promote local knowledge and empower farmers to take an active role in managing their genetic resources^70^.

## Conclusion

This study demonstrated that it was possible to cultivate wild ecotypes of *S. flexuosa* (L.) Medik. from seeds harvested in Northern Morocco. The agro-, morpho-, pheno-, and nutritional characteristics of these ecotypes grown on the experimental site are close to those reported in the literature for different varieties of *S. coronaria* (L.) Medik. that have been domesticated and selected for a long time. Four groups were distinguished on the basis mainly of the quantitative parameters of plant production, reproduction, and nutritional value. To get the highest protein and dry matter content to better use *S. flexuosa* (L.) Medik. in animal diet, cutting at the start of the flowering stage could be a compromise between dry matter yield, protein content, and digestibility. Future trials are needed to continue the domestication and the selection of *S. flexuosa* (L.) Medik., to spread this plant to the breeders and to conserve local genes in a seed bank. Moreover, climate change and recurrent droughts must be considered in the selection scheme.

## Data Availability

All data generated or analyzed during this study are included or specified in this published article. They are available from the corresponding author on reasonable request.
